# Morphea-like subcutaneous panniculitis-like T-cell lymphoma

**DOI:** 10.1016/j.jdcr.2023.12.025

**Published:** 2024-02-10

**Authors:** Keiko Tokuchi, Teruki Yanagi, Emi Inamura, Takuya Kawamura, Takashi Seo, Yasuyuki Fujita, Masao Nakagawa, Ikumi Kasahara, Yoshihiro Matsuno, Hideyuki Ujiie

**Affiliations:** a Department of Dermatology, Faculty of Medicine and Graduate School of Medicine, Hokkaido University, Sapporo, Japan; b Department of Dermatology, Sapporo City General Hospital, Sapporo, Japan; c Department of Hematology, Faculty of Medicine and Graduate School of Medicine, Hokkaido University, Sapporo, Japan; d Department of Hematology, Sapporo City General Hospital, Sapporo, Japan; e Department of Surgical Pathology, Hokkaido University Hospital, Sapporo, Japan

**Keywords:** cutaneous lymphoma, lupus erythematosus profundus, morphea, subcutaneous panniculitis-like T-cell lymphoma

## Introduction

Subcutaneous panniculitis-like T-cell lymphoma (SPTCL) is a primary cutaneous lymphoma that mainly affects the subcutaneous fat and accounts for approximately 1% to 2.3% of cutaneous lymphomas.^1^^,^^2^ The typical manifestations include multiple erythema nodosum-like subcutaneous nodules or plaques on the extremities. SPTCL is a lymphoma caused by αβ-type CD8-positive cytotoxic T cells infiltrating subcutaneous fat.^1^ In certain cases, SPTCL is challenging to differentiate from erythema nodosum, cellulitis, and lupus erythematosus profundus. Here, we present a case of SPTCL in which the initial clinicopathological findings resembled morphea.

## Report of a case

**Fig 1:**
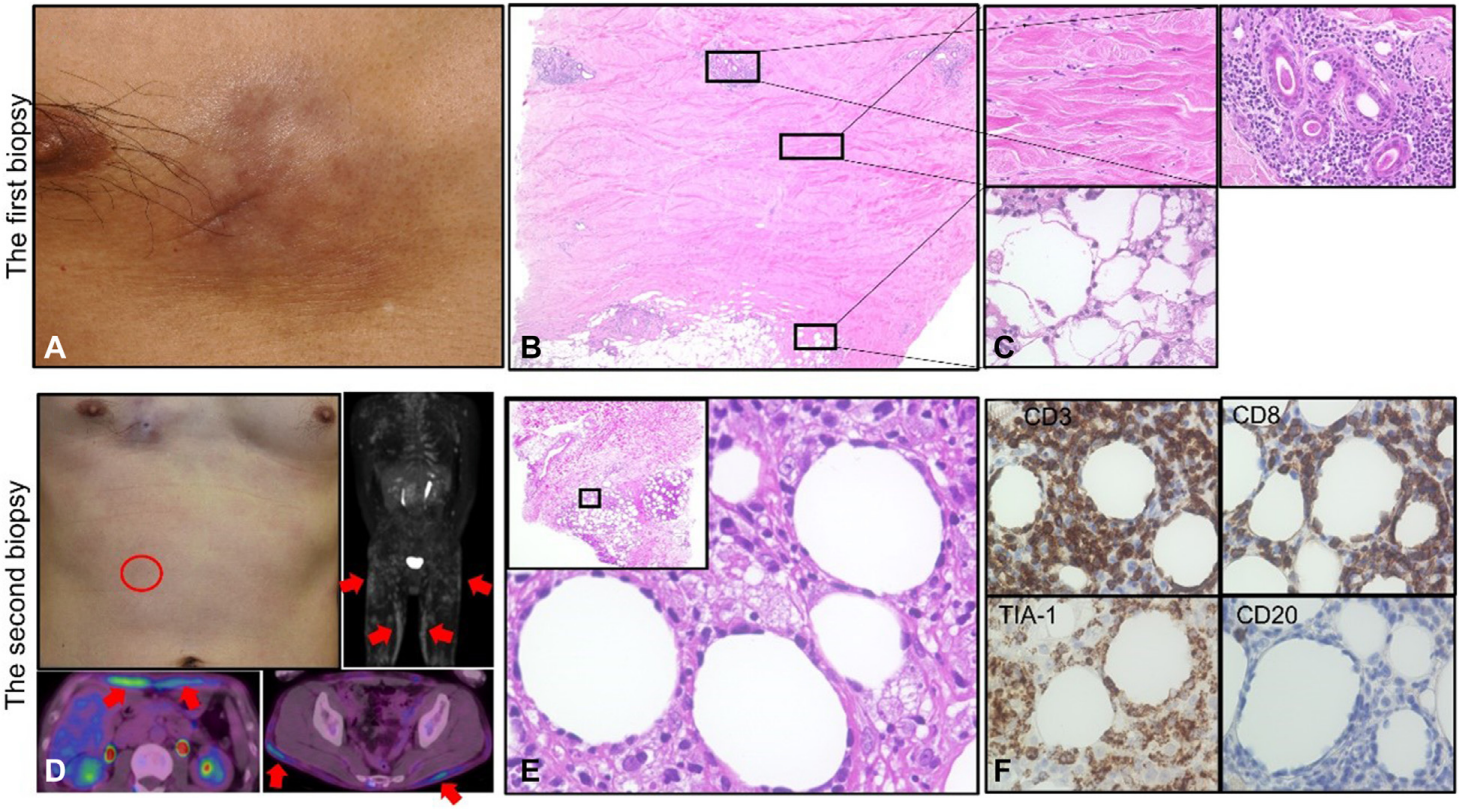
Clinical and histopathological findings of the first and second biopsy. **A,** The initial assessment reveals an indurated hyperpigmented plaque measuring 8 × 6 cm on the right chest. **B** and **C,** A skin biopsy reveals dermal and subcutaneous fat swelling with increased collagen fibers. The infiltration of dense mononuclear cells is present around the dermal appendages and subcutaneous fat without rimming. Additionally, fat necrosis is observed. (**B,** magnification ×40; **C,** magnification ×200 [*upper left*], and ×400 [*right* and *lower left*]). **D,** Multiple papules appear on the trunk and extremities (the *red circle* indicates the biopsy site). FDG-positron emission tomography computed tomography imaging exhibits multiple regions of FDG uptake in the subcutaneous and retroperitoneal adipose tissues throughout the body (*red arrows*). **E,** The second biopsy on one of the multiple erythematous areas with subcutaneous induration reveals dense lobular lymphoid infiltrates. Notably, adipocytes are surrounded by pleomorphic lymphocytes, which is known as “rimming” (magnification: ×40 [inset] and ×400). The squares in part labels **B** and **E** indicate that the enclosed regions are being magnified. **F,** The abnormal lymphocytes are positive for CD3, CD8, and T-cell intracellular antigen, but negative for CD20 (magnification ×400). *TIA-1*, T-cell intracellular antigen.

A 45-year-old male with a 1-year history of subcutaneous indurations was referred to our department. Initial examination revealed an 8 × 6 cm, markedly indurated hyperpigmented plaque on the right chest (Fig 1, *A*). A 2- × 1-cm subcutaneous plaque was also observed on the left chest. A skin biopsy specimen from the right chest revealed thick homogeneous increased collagen fibers in the dermis and subcutaneous fat (Fig 1, *B* and *C*). An infiltration of dense mononuclear cells was present around the dermal appendages and subcutaneous fat, accompanied by fat necrosis (Fig 1, *C*). The blood test results for complete blood count, antinuclear antibody (1:80), and soluble interleukin 2 receptor (225 U/ml) were all within normal ranges. Neither computed tomography from the neck to the pelvis nor gastrointestinal endoscopy showed any abnormalities. Although the presence of accompanying fat necrosis was atypical, he was initially diagnosed as morphea and administered prednisolone (PSL) (0.5 mg/kg/day). The symptoms improved, and PSL was gradually tapered. Two and a half years after the start of PSL, the patient noticed dyspnea, left mandibular swelling, headaches, and night sweats. Multiple red to brownish papules appeared on the trunk and extremities (Fig 1, *D*). A biopsy specimen from the erythema on the abdomen revealed inflammatory cells infiltrating subcutaneous fat, consisting of lymphocytes, plasma cells, and histiocytes accompanied by adipocyte rimming (Fig 1, *E*). The lymphocytes showed irregularly shaped nuclei and little to no infiltration in the dermis. Immunohistochemical analysis showed positivity for CD3, CD5, CD7, CD8, T-cell intracellular antigen, perforin, and T-cell receptor (TCR) βF1, but negativity for CD20, CD30, CD56, and TCR Cγ. In situ hybridization revealed the absence of EBV-encoded small RNA (Fig 1, *F*, Supplementary Fig 1, available via Mendeley at https://doi.org/10.17632/tpppbcwbwx.1). The Ki-67 labeling index was 74%. Although southern blot analyses of monoclonal rearrangement for TCR beta and gamma genes were negative, the final diagnosis of SPTCL was made given the overall clinical and histopathologic features. Lab tests revealed a white blood cell count of 6.1 × 10^9^/L (3.3-8.6 × 10^9^/L), a red blood cell count of 4.18 × 10^12^ (4.35-5.55 × 10^12^), hemoglobin 11.3 g/dl (13.7-16.8 g/dl), lactate dehydrogenase 970 U/L (124-222 U/L), and soluble interleukin 2 receptor 2408 U/ml (0-613 U/ml). Positron emission tomography computed tomography showed abnormal ^18^F-fluorodeoxyglucose uptake (standardized uptake value max: 8.363) in the subcutaneous and retroperitoneal adipose tissues throughout the body (Fig 1, *D*). Bone marrow aspiration tests ruled out hemophagocytic syndrome. The dosage of PSL was increased to 1 mg/day, but no improvement was observed. The patient underwent 1 course of systemic cyclophosphamide, doxorubicin, vincristine, and prednisone, followed by 5 courses of brentuximab vedotin plus cyclophosphamide, doxorubicin, and prednisone. Both the cutaneous lesions and the systemic symptoms resolved. He remains without evidence of recurrence 15 months after the completion of chemotherapy.

## Discussion

In SPTCL, the rimming of fat cells by atypical lymphocytes and fat necrosis are frequently observed. Immunohistochemistry usually shows the lymphoma cells to be positive for CD3, CD8, granzyme B, and T-cell intracellular antigen but negative for CD4 and CD56.^1^ TCR gene rearrangement studies are useful for excluding autoimmune diseases^3^ (Table I). The clinicopathologic picture evolves over time in SPTCL, so it can be difficult to diagnose on initial presentation. In the early phase of the present case, indurated plaques were observed mainly on the chest, but no obvious panniculitis-like skin manifestations were seen, such as tender indurated erythema and nodules, especially on the extremities. Also, the initial skin biopsies showed no clear evidence of malignant lymphoma, although atypical features such as fat necrosis were observed. In the later phase, clinical panniculitis-like manifestations became prominent, and the skin biopsy revealed the overt pathologic findings of SPTCL. There has been only 1 case report of SPTCL that was initially diagnosed as scleroderma. The patient presented with multiple subcutaneous lesions without systemic symptoms or serological abnormalities. Subsequent biopsies revealed the focal thickening of collagen fibers in the dermis and dense lymphoid cells infiltration of subcutaneous fat. The immunohistochemistry showed positive for CD8 antigen and cytotoxic molecules. Finally, a diagnosis of SPTCL was made.^4^ Additionally, a case of systemic scleroderma combined with T-cell lymphoma has been reported. Increased levels of cytokines (likely transforming growth factor β) produced by T-cell lymphoma cells have been considered responsible for the development of skin sclerosis.^5^ These studies suggest that some T-cell lymphomas can lead to scleroderma-like skin findings.

**Table I:** Summary of the features of subcutaneous panniculitis-like T-cell lymphoma, morphea, and lupus erythematosus profundus

	Subcutaneous T-cell lymphoma	Morphea	Lupus erythematosus profundus
Clinical features	Erythema nodosum-like appearance	Subcutaneous induration	Erythema nodosum-like appearance
Histology	Rimming of the adipocytes Clusters of pleomorphic lymphocytes Fibrinoid or coagulative fat necrosis	Thick collagen bundles Atrophy of dermal appendages	Clusters of plasmacytoid dendritic cells Mucinous changes Hyaline or lipomembranous fat necrosis
T-cell receptors	Rearrangement (+) or (−)	Rearrangement (−)	Rearrangement (−)

Lupus erythematosus profundus, an autoimmune disorder characterized by lobular panniculitis, is a differential diagnosis of SPTCL. “Ki-67 hotspots” with a >30% positive Ki-67 ratio have been reported to be a biomarker of SPTCL.^3^ In our case, the Ki-67 labeling index was 74% for the second biopsy, which is consistent with SPTCL.

Systemic lymphoma with secondary cutaneous involvement must be considered as a differential diagnosis.^6^ In this case, CD20 negativity ruled out the B-cell lineage. The histopathologic findings helped us rule out peripheral T-cell lymphoma not otherwise specified as a potential diagnosis. Natural killer/T-cell lymphomas typically express CD56 and are associated with Epstein-Barr Virus expression. The absence of CD30 rules out CD8^+^ systemic anaplastic large-cell lymphoma.

In summary, our patient initially presented with morphea-like skin lesions that gradually evolved into SPTCL over the course of more than a year. In SPTCL, systemic B-symptoms (fever, night sweats, and weight loss) are observed in 50% to 60% of cases, and patients may also present cytopenia and/or transaminitis.^7^^,^^8^ For patients with atypical morphea-like skin lesions who develop systemic symptoms or hematological abnormalities or who are resistant to typical therapies, repeated skin biopsies are recommended.

## Conflicts of interest

None disclosed.
